# A Case of Reflex Neuralgia

**Published:** 1875-05

**Authors:** 


*A Case of Reflex Neuralgia*, associated with Urethral Contractions and a rare form of Urinary Sinus, with a description of the Cold Water Coil. By Fessender N. Otis, M. D. Publishers: D. Appleton & Co., 549 and 551 Broadway, New York.

